# (4*R*,4a*S*,4b*S*,7*R*,10a*R*)-4-Hy­droxy-4a,7-dimethyl-2-(propan-2-yl)-1,4,4a,4b,5,6,7,8,10,10a-deca­hydro­phenanthren-1-one

**DOI:** 10.1107/S1600536811044540

**Published:** 2011-10-29

**Authors:** Ignez Caracelli, Julio Zukerman-Schpector, André T. Lousada Machado, Timothy J. Brocksom, M. Lúcia Ferreira, Edward R. T. Tiekink

**Affiliations:** a BioMat-Departamento de Física, Universidade Federal de São Carlos, CP 676, 13565-905, São Carlos, SP, Brazil; b Laboratório de Cristalografia, Estereodinâmica e Modelagem Molecular, Universidade Federal de São Carlos, Departamento de Química, CP 676, 13565-905, São Carlos, SP, Brazil; c Department of Chemistry, Universidade Federal de São Carlos, 13565-905 São Carlos, SP, Brazil; d Department of Chemistry, University of Malaya, 50603 Kuala Lumpur, Malaysia

## Abstract

In the title compound, C_19_H_28_O_2_, the *A* ring adopts a chair conformation, and each of the *B* and *C* rings adopts a distorted half-chair conformation with the methine C atom in the CH_2_C(H)C(=O) residue, common to both rings, lying 0.6397 (19) and 0.6328 (18) Å out of the approximate plane defined by the remaining five C atoms (r.m.s. deviations = 0.0791 and 0.0901 Å for rings *B* and *C*, respectively). Helical supra­molecular chains along the *a* axis mediated by hy­droxy–carbonyl O—H⋯O hydrogen bonds feature in the crystal packing.

## Related literature

For background to the biological activity of some diterpene compounds, see: Guo *et al.* (2011 ▶); Slusarczyk *et al.* (2011 ▶). For the synthesis, see: Ferreira (2002 ▶). For conformational analysis, see: Cremer & Pople (1975 ▶).
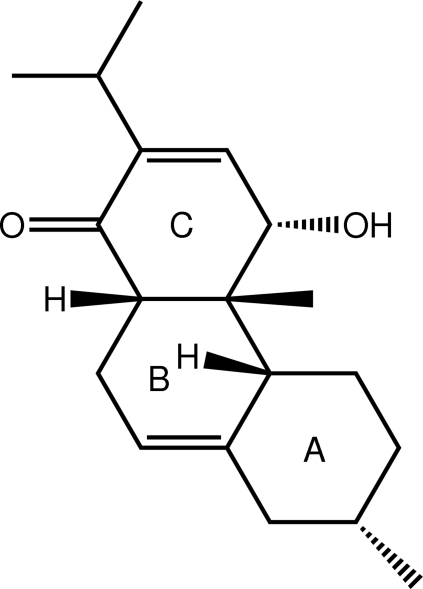


## Experimental

### 

#### Crystal data

C_19_H_28_O_2_
*M* *_r_* = 288.41
Orthorhombic, 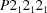

*a* = 7.3029 (9) Å
*b* = 13.211 (2) Å
*c* = 17.224 (3) Å
*V* = 1661.8 (4) Å^3^
*Z* = 4
Mo *K*α radiation
μ = 0.07 mm^−1^
*T* = 290 K
0.12 × 0.08 × 0.07 mm

#### Data collection

Enraf–Nonius CAD-4 MACH 3 diffractometer
3684 measured reflections
3243 independent reflections
2253 reflections with *I* > 2σ(*I*)
*R*_int_ = 0.031
3 standard reflections every 30 min  intensity decay: 1.1%

#### Refinement

*R*[*F* ^2^ > 2σ(*F* ^2^)] = 0.037
*wR*(*F* ^2^) = 0.113
*S* = 1.04
3243 reflections
195 parameters
H-atom parameters constrained
Δρ_max_ = 0.15 e Å^−3^
Δρ_min_ = −0.13 e Å^−3^

### 

Data collection: *CAD-4 Software* (Enraf–Nonius, 1989 ▶); cell refinement: *CAD-4 Software*; data reduction: *MolEN* (Fair, 1990 ▶); program(s) used to solve structure: *SIR92* (Altomare *et al.*, 1999 ▶); program(s) used to refine structure: *SHELXL97* (Sheldrick, 2008 ▶); molecular graphics: *ORTEP-3* (Farrugia, 1997 ▶), *DIAMOND* (Brandenburg, 2006 ▶) and *MarvinSketch* (Chemaxon, 2009 ▶); software used to prepare material for publication: *publCIF* (Westrip, 2010 ▶).

## Supplementary Material

Crystal structure: contains datablock(s) global, I. DOI: 10.1107/S1600536811044540/hg5120sup1.cif

Structure factors: contains datablock(s) I. DOI: 10.1107/S1600536811044540/hg5120Isup2.hkl

Supplementary material file. DOI: 10.1107/S1600536811044540/hg5120Isup3.cml

Additional supplementary materials:  crystallographic information; 3D view; checkCIF report

## Figures and Tables

**Table 1:** Hydrogen-bond geometry (Å, °)

*D*—H⋯*A*	*D*—H	H⋯*A*	*D*⋯*A*	*D*—H⋯*A*
O2—H2*o*⋯O1^i^	0.82	2.01	2.805 (2)	162

Symmetry code: (i) 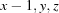.

**Fig. 1.**
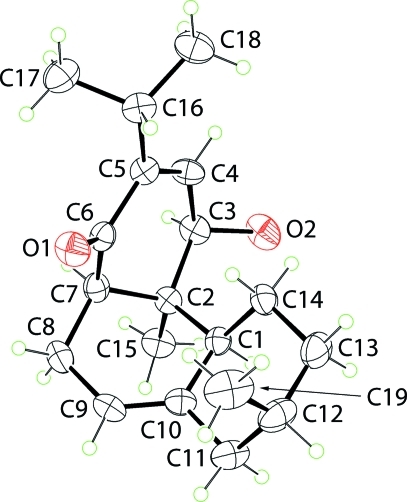
The molecular structure of compound (I) showing atom labelling scheme and displacement ellipsoids at the 30% probability level (arbitrary spheres for the H atoms).

**Fig. 2.**
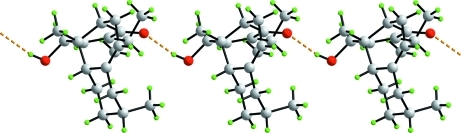
A view of the helical supramolecular chain along the a axis in (I). The hydroxy-O—H···O(carbonyl) hydrogen bonds are represented by orange dashed lines.

## supplementary crystallographic information

### Comment

Natural diterpenes exhibit a wide range of biological activities such as neuroprotectives (Guo *et al.* 2011) and as antiplasmodials and antitrypanocidals (Slusarczyk *et al.* 2011). While aiming at the synthesis of some hydrophenanthrene diterpenes, a series of new intermediates were obtained and among them, was the title compound (Ferreira, 2002), (I), which has been characterized crystallographically.

The A ring has a chair conformation. Each of the B and C rings presents a distorted half-chair conformation with atom C7, common to both rings, lying 0.6397 (19) and 0.6328 (18) Å, for B and C, respectively, out of the approximate plane defined by the remaining five C atoms (r.m.s. deviations = 0.0791 and 0.0901, respectively). The ring puckering parameters are: q_2_ = 0.003 (2), 0.360 (2), 0.363 (2) Å; q_3_ = 0.570 (2), -0.315 (2), -0.332 (2) Å; QT = 0.570 (2), 0.478 (2), 0.492 (2) Å; and θ = 1.7 (2), 131.1 (2), 132.5 (2)°, for rings A, B and C, respectively (Cremer & Pople, 1975).

In the crystal packing, the molecules are linked through O–H···O hydrogen bonds to form supramolecular helical chains along the *a* axis, Fig. 2 and Table 1.

### Experimental

The detailed synthesis of the title compound is described in a Ph.D. thesis (Ferreira, 2002). Crystals were grown by slow evaporation from its hexane solution held at 293 K; *M*.pt: 466.2–468.1 K. ^1^H-NMR (CDCl_3_, 400 MHz): δ (p.p.m.): 6.44 (d, 1H, J = 3.8 Hz); 5.26 (s, 1H); 4.58 (s, 1H); 2.81–2.87 (m, 1H); 2.86–2.92 (m, 1H); 2.24 (d, 1H, J = 6.4 Hz); 2.10–2.26 (m, 2H); 1.90–2.10 (m, 2H); 1.82–2.10 (m, 1H); 1.37–1.54 (m, 4H); 1.27 (s, 3H); 1.04 (d, 3H, J = 6.8 Hz); 1.01 (d, 3H, J = 6.8 Hz); 0.73 (d, 3H, J = 6.8 Hz); δ(OH) not obs. ^13^C (CDCl_3_, 100 MHz) δ (p.p.m.): 197.9; 144.4; 142.7; 135.5; 117.2; 76.1; 49.6; 49.5; 45.9; 44.6; 35.7; 35.6; 33.6; 23.6; 22.3; 21.7; 21.5; 20.2; 20.0. Analysis found: C 78.99, H 9.77%. C_19_H_28_O_2_ requires: C 79.12, H 9.79%.

### Refinement

The H atoms were geometrically placed (C—H = 0.93–0.98 Å; O—H = 0.82 Å) and refined as riding with *U_iso_*(H) = 1.2*U_eq_*(C) and *U_iso_*(H) = 1.5*U_eq_*(methyl-C,*O*). The absolute structure was based on that of a starting material used in the synthesis (Ferreira, 2002).

### Crystal data

**Table:**

C_19_H_28_O_2_	*F*(000) = 632
*M**_r_* = 288.41	*D*_x_ = 1.153 Mg m^−^^3^
Orthorhombic, *P*2_1_2_1_2_1_	Mo *K*α radiation, λ = 0.71073 Å
Hall symbol: P 2ac 2ab	Cell parameters from 25 reflections
*a* = 7.3029 (9) Å	θ = 9.2–14.3°
*b* = 13.211 (2) Å	µ = 0.07 mm^−^^1^
*c* = 17.224 (3) Å	*T* = 290 K
*V* = 1661.8 (4) Å^3^	Irregular, colourless
*Z* = 4	0.12 × 0.08 × 0.07 mm

### Data collection

**Table:**

Enraf–Nonius CAD-4 MACH 3 diffractometer	*R*_int_ = 0.031
Radiation source: fine-focus sealed tube	θ_max_ = 26.0°, θ_min_ = 1.9°
graphite	*h* = 0→8
ω/–2θ scans	*k* = 0→16
3684 measured reflections	*l* = −21→21
3243 independent reflections	3 standard reflections every 30 min
2253 reflections with *I* > 2σ(*I*)	intensity decay: 1.1%

### Refinement

**Table:**

Refinement on *F*^2^	Primary atom site location: structure-invariant direct methods
Least-squares matrix: full	Secondary atom site location: difference Fourier map
*R*[*F*^2^ > 2σ(*F*^2^)] = 0.037	Hydrogen site location: inferred from neighbouring sites
*wR*(*F*^2^) = 0.113	H-atom parameters constrained
*S* = 1.04	*w* = 1/[σ^2^(*F*_o_^2^) + (0.0653*P*)^2^ + 0.0421*P*] where *P* = (*F*_o_^2^ + 2*F*_c_^2^)/3
3243 reflections	(Δ/σ)_max_ < 0.001
195 parameters	Δρ_max_ = 0.15 e Å^−^^3^
0 restraints	Δρ_min_ = −0.13 e Å^−^^3^

### Special details

**Table:**

Geometry. All s.u.'s (except the s.u. in the dihedral angle between two l.s. planes) are estimated using the full covariance matrix. The cell s.u.'s are taken into account individually in the estimation of s.u.'s in distances, angles and torsion angles; correlations between s.u.'s in cell parameters are only used when they are defined by crystal symmetry. An approximate (isotropic) treatment of cell s.u.'s is used for estimating s.u.'s involving l.s. planes.
Refinement. Refinement of *F*^2^ against ALL reflections. The weighted *R*-factor *wR* and goodness of fit *S* are based on *F*^2^, conventional *R*-factors *R* are based on *F*, with *F* set to zero for negative *F*^2^. The threshold expression of *F*^2^ > 2σ(*F*^2^) is used only for calculating *R*-factors(gt) *etc*. and is not relevant to the choice of reflections for refinement. *R*-factors based on *F*^2^ are statistically about twice as large as those based on *F*, and *R*- factors based on ALL data will be even larger.

### Fractional atomic coordinates and isotropic or equivalent isotropic displacement parameters (Å^2^)

**Table:**

	*x*	*y*	*z*	*U*_iso_*/*U*_eq_	
C1	0.6835 (3)	0.92045 (14)	0.83208 (11)	0.0386 (4)	
H1	0.5721	0.9042	0.8613	0.046*	
C2	0.6251 (2)	0.99993 (14)	0.77072 (11)	0.0378 (4)	
C3	0.5391 (2)	0.95441 (16)	0.69613 (12)	0.0457 (5)	
H3	0.4762	1.0096	0.6691	0.055*	
C4	0.6752 (3)	0.91091 (16)	0.64072 (11)	0.0448 (5)	
H4	0.6311	0.8659	0.6036	0.054*	
C5	0.8545 (2)	0.93064 (15)	0.63955 (10)	0.0387 (4)	
C6	0.9268 (2)	0.99737 (15)	0.70073 (11)	0.0382 (4)	
C7	0.7918 (3)	1.06323 (13)	0.74402 (11)	0.0417 (5)	
H7	0.7468	1.1144	0.7075	0.050*	
C8	0.8794 (3)	1.11916 (16)	0.81222 (12)	0.0498 (5)	
H8A	0.9985	1.1447	0.7967	0.060*	
H8B	0.8036	1.1766	0.8263	0.060*	
C9	0.9011 (3)	1.05182 (15)	0.88050 (12)	0.0471 (5)	
H9	0.9814	1.0725	0.9193	0.056*	
C10	0.8151 (3)	0.96451 (16)	0.89061 (11)	0.0415 (4)	
C11	0.8553 (3)	0.89695 (17)	0.95906 (12)	0.0571 (6)	
H11A	0.7435	0.8853	0.9881	0.068*	
H11B	0.9424	0.9300	0.9932	0.068*	
C12	0.9340 (4)	0.79528 (18)	0.93174 (14)	0.0613 (7)	
H12	0.9403	0.7499	0.9767	0.074*	
C13	0.8019 (4)	0.74886 (17)	0.87258 (13)	0.0626 (7)	
H13A	0.8539	0.6862	0.8532	0.075*	
H13B	0.6878	0.7324	0.8985	0.075*	
C14	0.7615 (3)	0.81779 (14)	0.80467 (12)	0.0462 (5)	
H14A	0.6739	0.7852	0.7705	0.055*	
H14B	0.8731	0.8292	0.7755	0.055*	
C15	0.4816 (3)	1.06970 (16)	0.80839 (13)	0.0520 (5)	
H15A	0.4561	1.1254	0.7743	0.078*	
H15B	0.5282	1.0950	0.8568	0.078*	
H15C	0.3711	1.0322	0.8176	0.078*	
C16	0.9912 (3)	0.88187 (16)	0.58484 (11)	0.0439 (5)	
H16	1.0830	0.8479	0.6172	0.053*	
C17	1.0923 (3)	0.95967 (18)	0.53606 (13)	0.0618 (6)	
H17A	1.1538	1.0067	0.5696	0.093*	
H17B	1.0064	0.9954	0.5040	0.093*	
H17C	1.1806	0.9260	0.5038	0.093*	
C18	0.9070 (3)	0.80139 (17)	0.53282 (14)	0.0597 (6)	
H18A	0.8167	0.8319	0.4998	0.090*	
H18B	0.8501	0.7503	0.5643	0.090*	
H18C	1.0009	0.7711	0.5015	0.090*	
C19	1.1271 (4)	0.8076 (2)	0.89947 (16)	0.0762 (8)	
H19A	1.1246	0.8534	0.8562	0.114*	
H19B	1.1724	0.7430	0.8827	0.114*	
H19C	1.2058	0.8343	0.9392	0.114*	
O1	1.09041 (18)	1.00004 (12)	0.71585 (9)	0.0548 (4)	
O2	0.4059 (2)	0.87899 (12)	0.71243 (11)	0.0637 (5)	
H2o	0.3032	0.9036	0.7089	0.096*	

### Atomic displacement parameters (Å^2^)

**Table:**

	*U*^11^	*U*^22^	*U*^33^	*U*^12^	*U*^13^	*U*^23^
C1	0.0338 (9)	0.0390 (10)	0.0431 (10)	0.0005 (8)	0.0042 (8)	0.0009 (8)
C2	0.0291 (9)	0.0410 (10)	0.0433 (10)	0.0040 (8)	−0.0015 (8)	−0.0020 (9)
C3	0.0289 (9)	0.0551 (12)	0.0531 (11)	0.0041 (8)	−0.0081 (8)	−0.0028 (10)
C4	0.0362 (10)	0.0579 (13)	0.0403 (10)	0.0065 (9)	−0.0090 (8)	−0.0087 (10)
C5	0.0311 (10)	0.0456 (10)	0.0395 (10)	0.0037 (8)	−0.0032 (7)	0.0031 (9)
C6	0.0313 (9)	0.0415 (10)	0.0419 (10)	−0.0020 (8)	−0.0019 (8)	0.0072 (9)
C7	0.0419 (11)	0.0357 (10)	0.0476 (11)	0.0007 (9)	−0.0046 (8)	0.0046 (9)
C8	0.0495 (12)	0.0389 (10)	0.0611 (13)	−0.0058 (10)	−0.0002 (10)	−0.0079 (10)
C9	0.0450 (11)	0.0497 (12)	0.0465 (11)	0.0010 (10)	−0.0085 (9)	−0.0142 (9)
C10	0.0393 (10)	0.0458 (11)	0.0393 (10)	0.0087 (9)	0.0003 (8)	−0.0045 (9)
C11	0.0652 (15)	0.0656 (14)	0.0404 (11)	0.0150 (12)	0.0001 (10)	0.0004 (10)
C12	0.0764 (16)	0.0607 (14)	0.0467 (12)	0.0222 (13)	0.0046 (11)	0.0148 (11)
C13	0.0857 (18)	0.0395 (12)	0.0626 (14)	0.0097 (12)	0.0142 (13)	0.0084 (11)
C14	0.0527 (11)	0.0352 (10)	0.0507 (11)	−0.0007 (9)	−0.0008 (10)	−0.0012 (9)
C15	0.0426 (11)	0.0572 (12)	0.0563 (12)	0.0147 (10)	−0.0011 (10)	−0.0066 (11)
C16	0.0340 (10)	0.0536 (12)	0.0440 (11)	0.0109 (10)	−0.0015 (9)	−0.0001 (9)
C17	0.0585 (14)	0.0705 (15)	0.0565 (13)	0.0026 (13)	0.0138 (12)	0.0031 (12)
C18	0.0542 (13)	0.0635 (14)	0.0615 (14)	0.0150 (12)	−0.0001 (12)	−0.0155 (11)
C19	0.0692 (17)	0.0917 (19)	0.0677 (15)	0.0340 (16)	−0.0017 (13)	0.0006 (15)
O1	0.0310 (7)	0.0714 (10)	0.0622 (10)	−0.0043 (7)	−0.0035 (7)	−0.0086 (8)
O2	0.0282 (7)	0.0769 (11)	0.0861 (12)	−0.0086 (8)	−0.0018 (8)	−0.0182 (9)

### Geometric parameters (Å, °)

**Table:**

C1—C10	1.510 (3)	C11—H11B	0.9700
C1—C14	1.545 (3)	C12—C19	1.524 (4)
C1—C2	1.550 (3)	C12—C13	1.532 (4)
C1—H1	0.9800	C12—H12	0.9800
C2—C15	1.539 (3)	C13—C14	1.511 (3)
C2—C7	1.547 (3)	C13—H13A	0.9700
C2—C3	1.551 (3)	C13—H13B	0.9700
C3—O2	1.421 (3)	C14—H14A	0.9700
C3—C4	1.493 (3)	C14—H14B	0.9700
C3—H3	0.9800	C15—H15A	0.9600
C4—C5	1.335 (3)	C15—H15B	0.9600
C4—H4	0.9300	C15—H15C	0.9600
C5—C6	1.472 (3)	C16—C17	1.519 (3)
C5—C16	1.517 (3)	C16—C18	1.520 (3)
C6—O1	1.223 (2)	C16—H16	0.9800
C6—C7	1.512 (3)	C17—H17A	0.9600
C7—C8	1.528 (3)	C17—H17B	0.9600
C7—H7	0.9800	C17—H17C	0.9600
C8—C9	1.483 (3)	C18—H18A	0.9600
C8—H8A	0.9700	C18—H18B	0.9600
C8—H8B	0.9700	C18—H18C	0.9600
C9—C10	1.325 (3)	C19—H19A	0.9600
C9—H9	0.9300	C19—H19B	0.9600
C10—C11	1.507 (3)	C19—H19C	0.9600
C11—C12	1.535 (3)	O2—H2o	0.8200
C11—H11A	0.9700		
			
C10—C1—C14	107.93 (16)	H11A—C11—H11B	108.1
C10—C1—C2	111.68 (15)	C19—C12—C13	112.5 (2)
C14—C1—C2	119.21 (15)	C19—C12—C11	111.4 (2)
C10—C1—H1	105.7	C13—C12—C11	108.6 (2)
C14—C1—H1	105.7	C19—C12—H12	108.1
C2—C1—H1	105.7	C13—C12—H12	108.1
C15—C2—C7	109.71 (16)	C11—C12—H12	108.1
C15—C2—C1	107.80 (15)	C14—C13—C12	113.36 (19)
C7—C2—C1	110.64 (15)	C14—C13—H13A	108.9
C15—C2—C3	107.81 (15)	C12—C13—H13A	108.9
C7—C2—C3	106.38 (15)	C14—C13—H13B	108.9
C1—C2—C3	114.42 (16)	C12—C13—H13B	108.9
O2—C3—C4	108.20 (17)	H13A—C13—H13B	107.7
O2—C3—C2	112.67 (17)	C13—C14—C1	111.38 (16)
C4—C3—C2	114.15 (15)	C13—C14—H14A	109.4
O2—C3—H3	107.1	C1—C14—H14A	109.4
C4—C3—H3	107.1	C13—C14—H14B	109.4
C2—C3—H3	107.1	C1—C14—H14B	109.4
C5—C4—C3	125.97 (18)	H14A—C14—H14B	108.0
C5—C4—H4	117.0	C2—C15—H15A	109.5
C3—C4—H4	117.0	C2—C15—H15B	109.5
C4—C5—C6	117.27 (17)	H15A—C15—H15B	109.5
C4—C5—C16	124.90 (18)	C2—C15—H15C	109.5
C6—C5—C16	117.57 (16)	H15A—C15—H15C	109.5
O1—C6—C5	121.36 (18)	H15B—C15—H15C	109.5
O1—C6—C7	121.03 (18)	C5—C16—C17	112.10 (17)
C5—C6—C7	117.61 (15)	C5—C16—C18	113.38 (17)
C6—C7—C8	112.58 (17)	C17—C16—C18	110.12 (18)
C6—C7—C2	110.42 (15)	C5—C16—H16	107.0
C8—C7—C2	111.26 (16)	C17—C16—H16	107.0
C6—C7—H7	107.4	C18—C16—H16	107.0
C8—C7—H7	107.4	C16—C17—H17A	109.5
C2—C7—H7	107.4	C16—C17—H17B	109.5
C9—C8—C7	111.36 (16)	H17A—C17—H17B	109.5
C9—C8—H8A	109.4	C16—C17—H17C	109.5
C7—C8—H8A	109.4	H17A—C17—H17C	109.5
C9—C8—H8B	109.4	H17B—C17—H17C	109.5
C7—C8—H8B	109.4	C16—C18—H18A	109.5
H8A—C8—H8B	108.0	C16—C18—H18B	109.5
C10—C9—C8	125.16 (18)	H18A—C18—H18B	109.5
C10—C9—H9	117.4	C16—C18—H18C	109.5
C8—C9—H9	117.4	H18A—C18—H18C	109.5
C9—C10—C11	121.7 (2)	H18B—C18—H18C	109.5
C9—C10—C1	123.35 (18)	C12—C19—H19A	109.5
C11—C10—C1	114.70 (18)	C12—C19—H19B	109.5
C10—C11—C12	110.57 (17)	H19A—C19—H19B	109.5
C10—C11—H11A	109.5	C12—C19—H19C	109.5
C12—C11—H11A	109.5	H19A—C19—H19C	109.5
C10—C11—H11B	109.5	H19B—C19—H19C	109.5
C12—C11—H11B	109.5	C3—O2—H2o	109.5
			
C10—C1—C2—C15	−77.5 (2)	C3—C2—C7—C6	−58.80 (19)
C14—C1—C2—C15	155.54 (17)	C15—C2—C7—C8	59.1 (2)
C10—C1—C2—C7	42.5 (2)	C1—C2—C7—C8	−59.7 (2)
C14—C1—C2—C7	−84.5 (2)	C3—C2—C7—C8	175.44 (16)
C10—C1—C2—C3	162.63 (15)	C6—C7—C8—C9	−79.0 (2)
C14—C1—C2—C3	35.6 (2)	C2—C7—C8—C9	45.5 (2)
C15—C2—C3—O2	−73.6 (2)	C7—C8—C9—C10	−17.0 (3)
C7—C2—C3—O2	168.73 (15)	C8—C9—C10—C11	175.48 (19)
C1—C2—C3—O2	46.3 (2)	C8—C9—C10—C1	1.1 (3)
C15—C2—C3—C4	162.42 (17)	C14—C1—C10—C9	118.6 (2)
C7—C2—C3—C4	44.8 (2)	C2—C1—C10—C9	−14.3 (3)
C1—C2—C3—C4	−77.7 (2)	C14—C1—C10—C11	−56.2 (2)
O2—C3—C4—C5	−144.8 (2)	C2—C1—C10—C11	170.94 (16)
C2—C3—C4—C5	−18.5 (3)	C9—C10—C11—C12	−116.8 (2)
C3—C4—C5—C6	3.3 (3)	C1—C10—C11—C12	58.0 (3)
C3—C4—C5—C16	177.17 (19)	C10—C11—C12—C19	70.1 (3)
C4—C5—C6—O1	162.0 (2)	C10—C11—C12—C13	−54.3 (3)
C16—C5—C6—O1	−12.4 (3)	C19—C12—C13—C14	−68.0 (3)
C4—C5—C6—C7	−18.4 (3)	C11—C12—C13—C14	55.8 (3)
C16—C5—C6—C7	167.19 (17)	C12—C13—C14—C1	−56.9 (3)
O1—C6—C7—C8	−7.3 (3)	C10—C1—C14—C13	54.0 (2)
C5—C6—C7—C8	173.06 (16)	C2—C1—C14—C13	−177.28 (19)
O1—C6—C7—C2	−132.3 (2)	C4—C5—C16—C17	121.9 (2)
C5—C6—C7—C2	48.0 (2)	C6—C5—C16—C17	−64.2 (2)
C15—C2—C7—C6	−175.15 (16)	C4—C5—C16—C18	−3.5 (3)
C1—C2—C7—C6	66.04 (19)	C6—C5—C16—C18	170.39 (18)

### Hydrogen-bond geometry (Å, °)

**Table:**

*D*—H···*A*	*D*—H	H···*A*	*D*···*A*	*D*—H···*A*
O2—H2o···O1^i^	0.82	2.01	2.805 (2)	162

Symmetry codes: (i) *x*−1, *y*, *z*.
